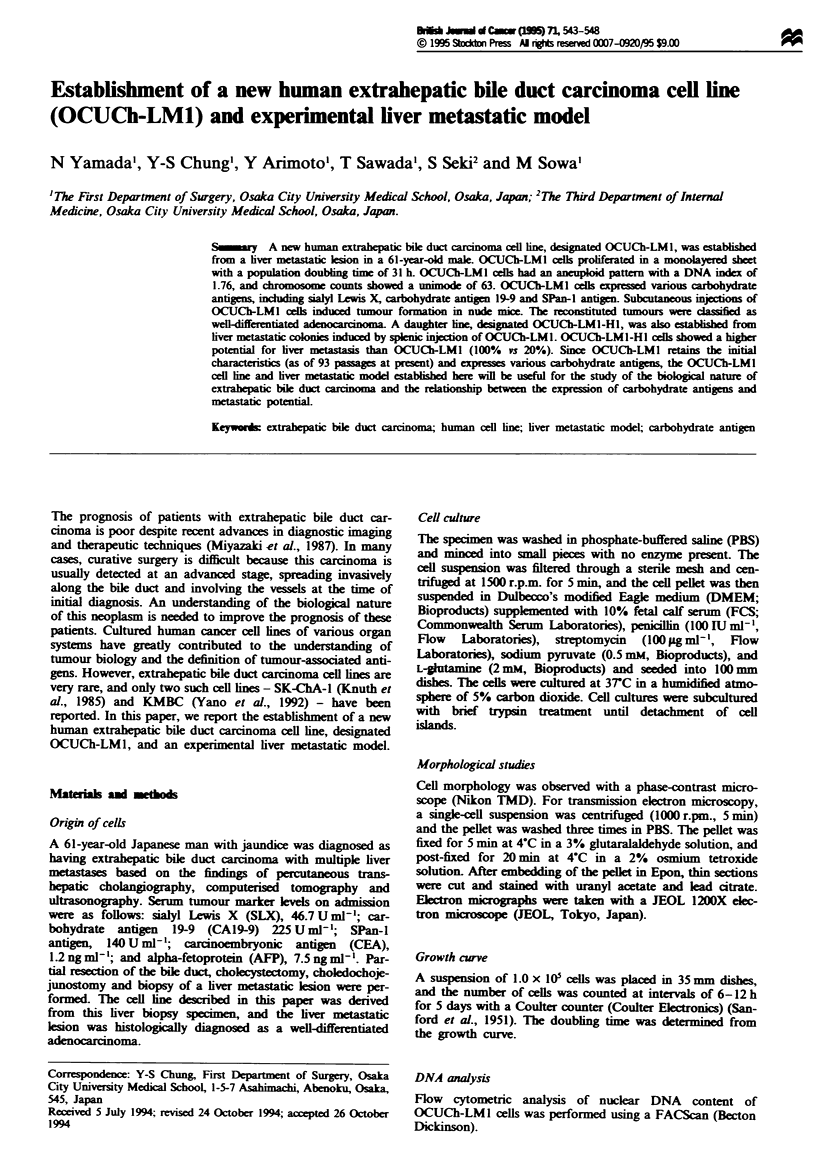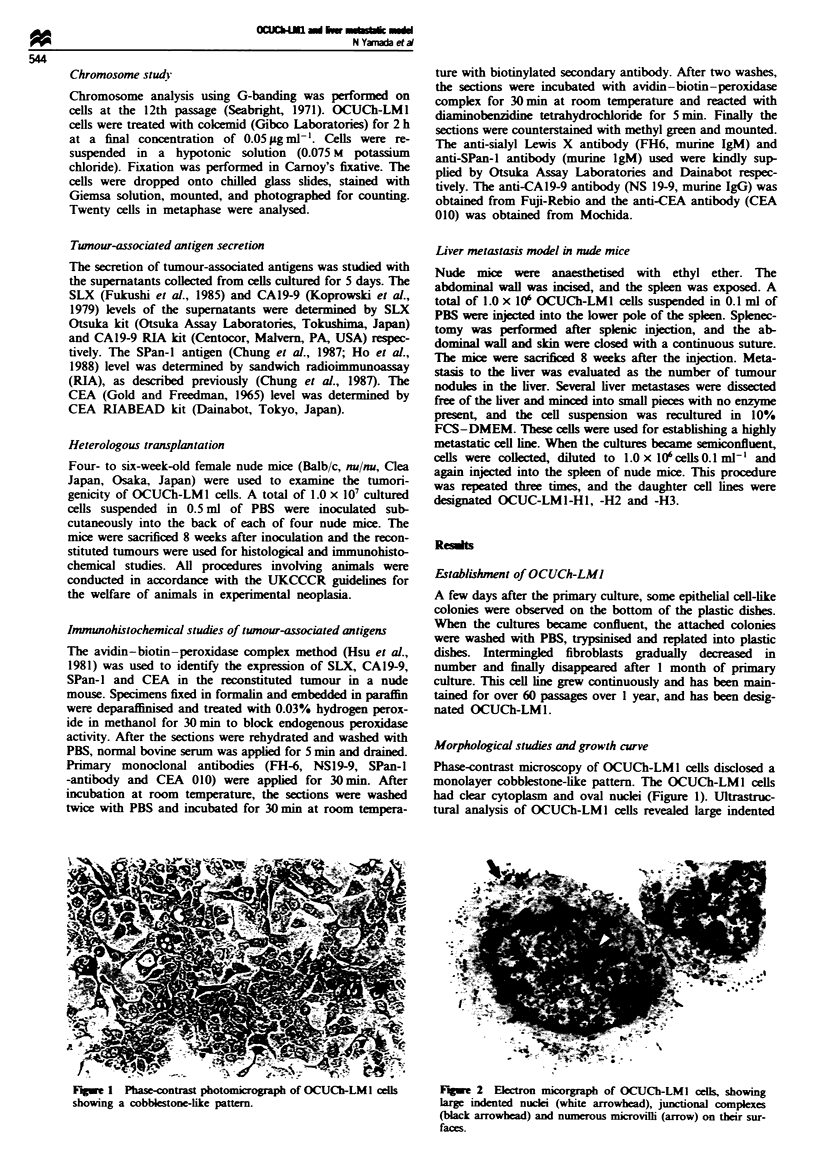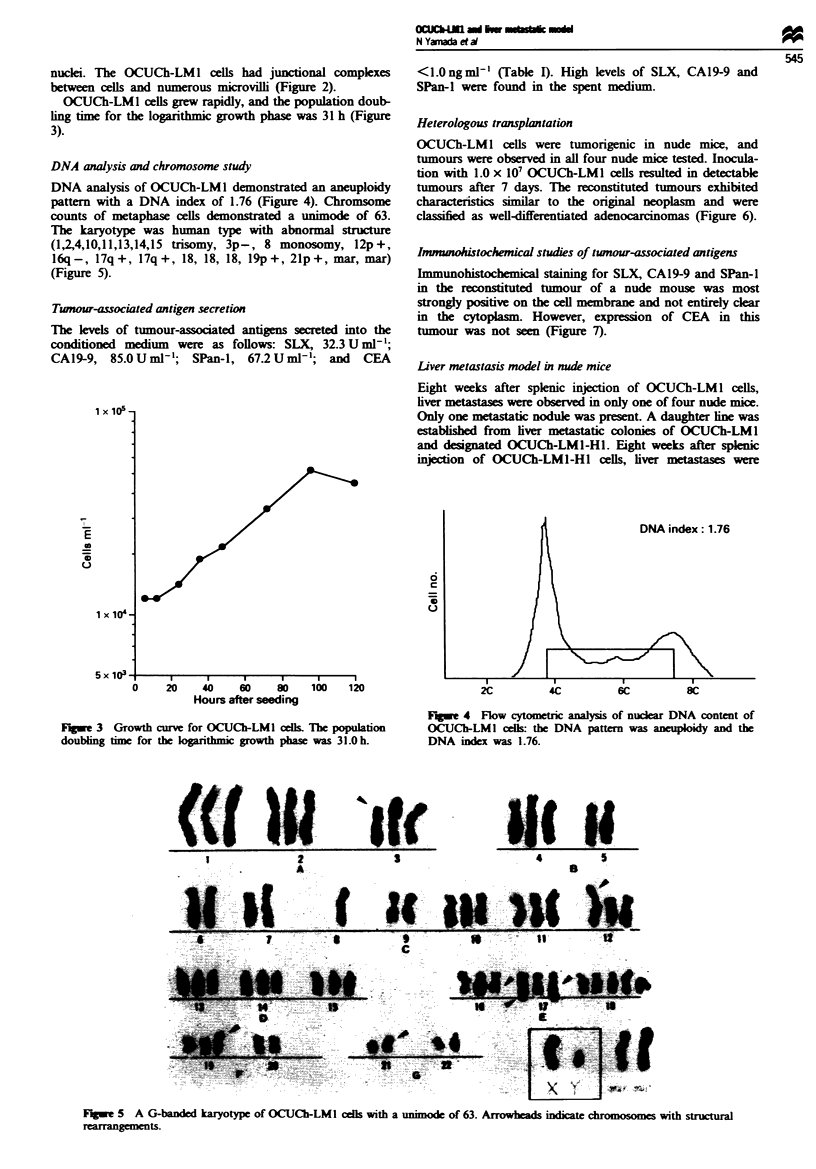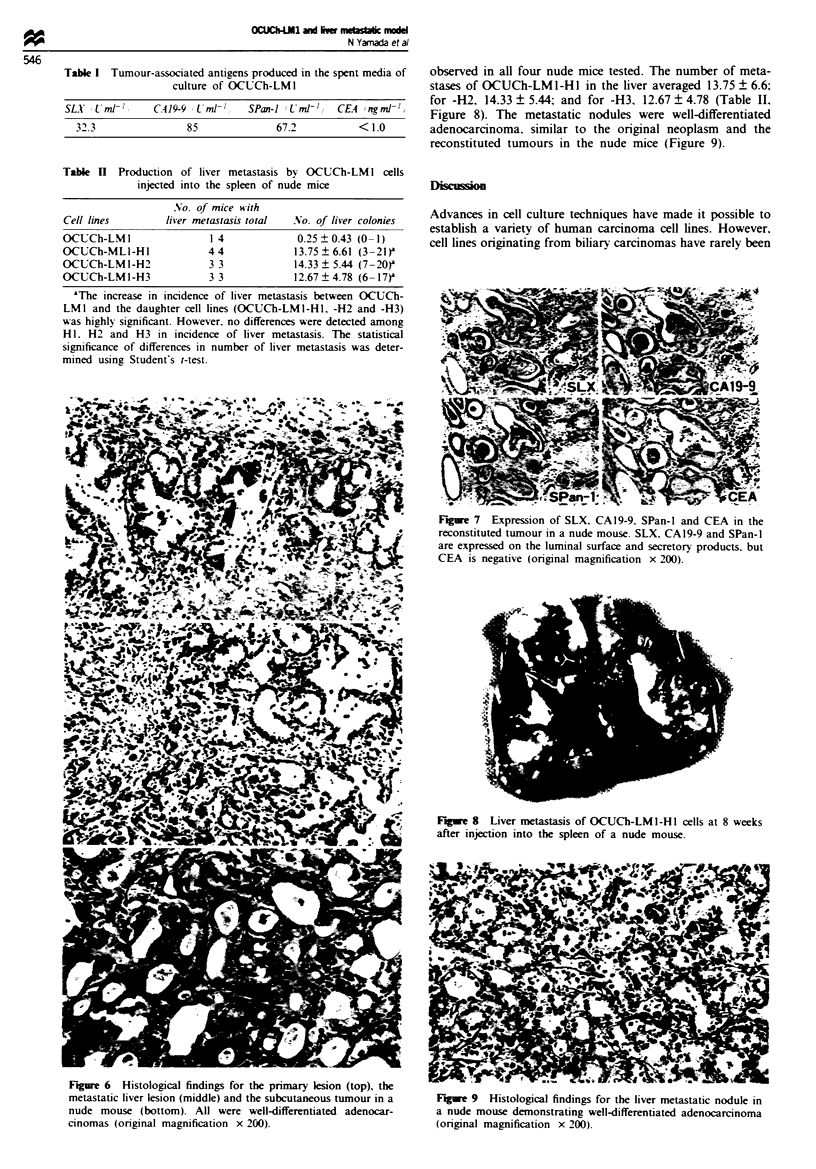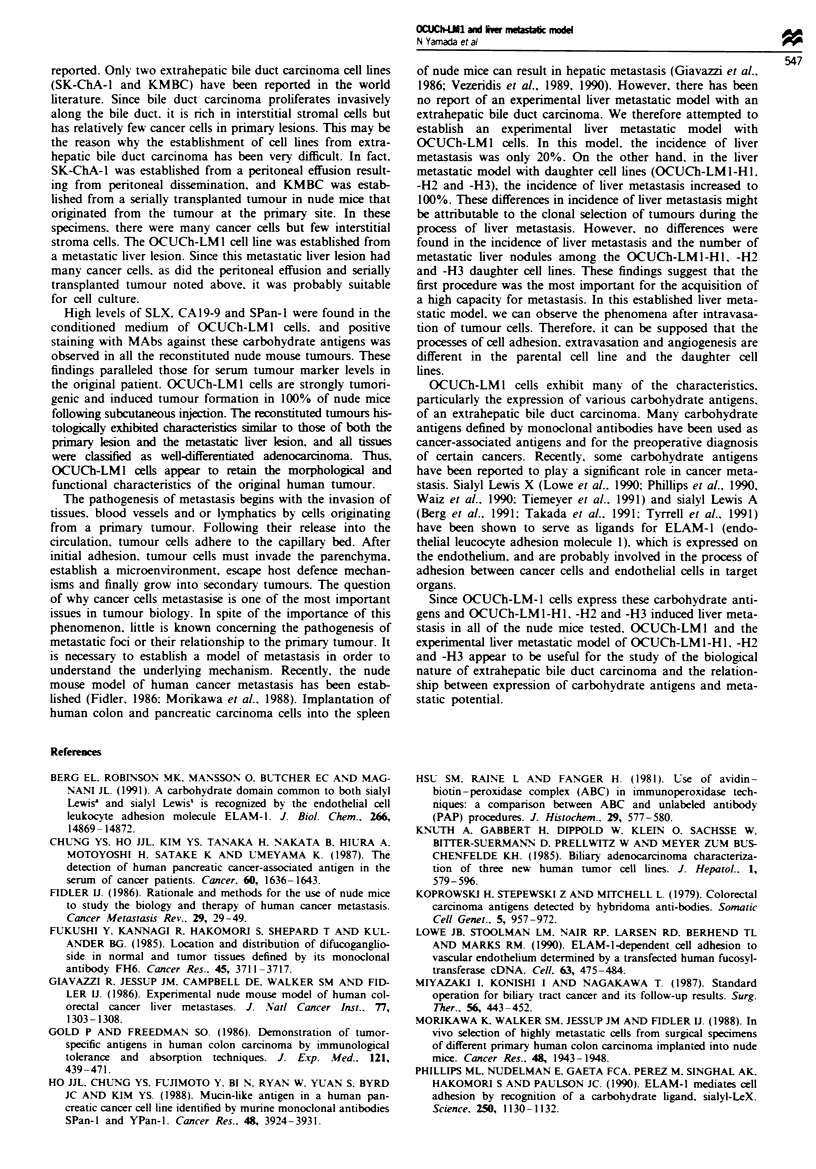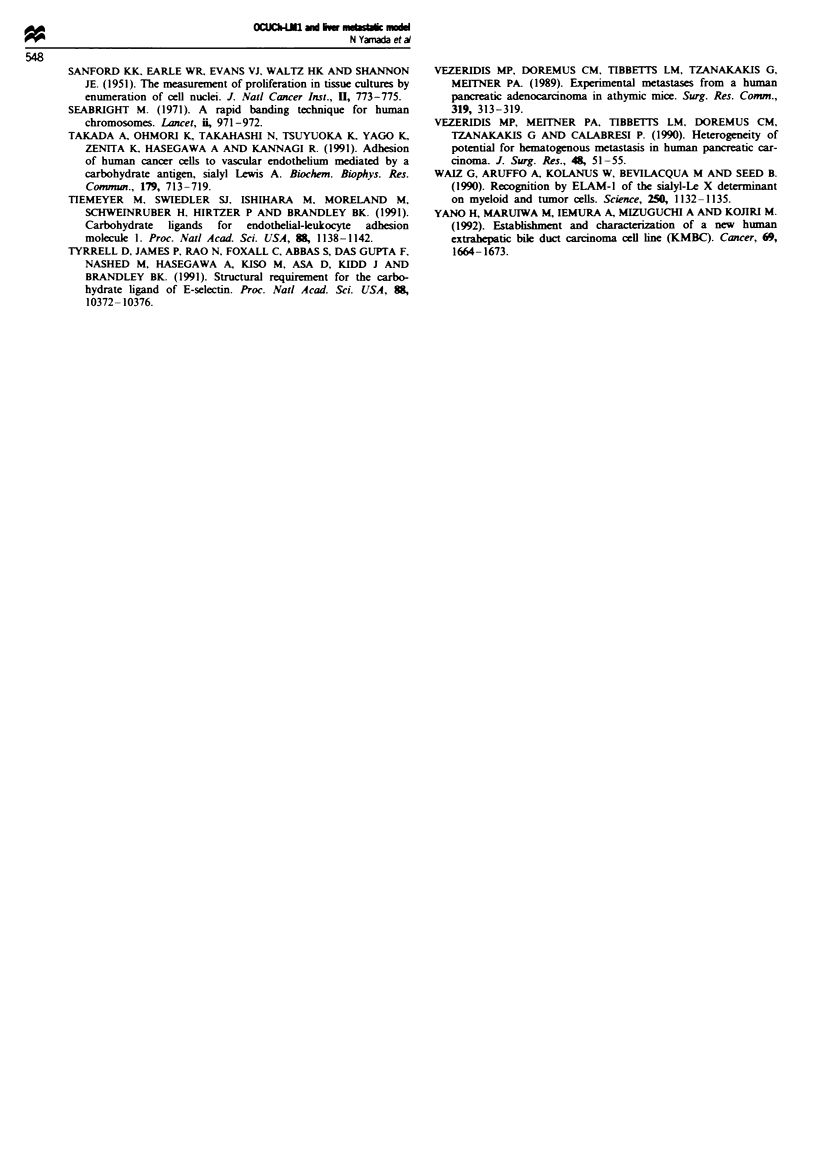# Establishment of a new human extrahepatic bile duct carcinoma cell line (OCUCh-LM1) and experimental liver metastatic model.

**DOI:** 10.1038/bjc.1995.107

**Published:** 1995-03

**Authors:** N. Yamada, Y. S. Chung, Y. Arimoto, T. Sawada, S. Seki, M. Sowa

**Affiliations:** First Department of Surgery, Osaka City University Medical School, Japan.

## Abstract

**Images:**


© 1995 Stockton Press All rights reserved 0007-0920/95 $9.00

### Establishment of a new human extrahepatic bile duct carcinoma cell line (OCUCh-LM1) and experimental liver metastatic model

N Yamada[1], Y-S Chung[1], Y Arimoto[1], T Sawada[1], S Seki[2] and M Sowa[1]

[1]*The First Department of Surgery, Osaka City University Medical School, Osaka, Japan;* [2]*The Third Department of Internal Medicine, Osaka City University Medical School, Osaka, Japan.*

**Summary** A new human extrahepatic bile duct carcinoma cell line, designated OCUCh-LM1, was established from a liver metastatic lesion in a 61-year-old male. OCUCh-LM1 cells proliferated in a monolayered sheet with a population doubling time of 31 h. OCUCh-LM1 cells had an aneuploid pattern with a DNA index of 1.76, and chromosome counts showed a unimode of 63. OCUCh-LM1 cells expressed various carbohydrate antigens, including sialyl Lewis X, carbohydrate antigen 19-9 and SPan-1 antigen. Subcutaneous injections of OCUCh-LM1 cells induced tumour formation in nude mice. The reconstituted tumours were classified as well-differentiated adenocarcinoma. A daughter line, designated OCUCh-LM1-H1, was also established from liver metastatic colonies induced by splenic injection of OCUCh-LM1. OCUCh-LM1-H1 cells showed a higher potential for liver metastasis than OCUCh-LM1 (100% *vs* 20%). Since OCUCh-LM1 retains the initial characteristics (as of 93 passages at present) and expresses various carbohydrate antigens, the OCUCh-LM1 cell line and liver metastatic model established here will be useful for the study of the biological nature of extrahepatic bile duct carcinoma and the relationship between the expression of carbohydrate antigens and metastatic potential.

**Keywords:** extrahepatic bile duct carcinoma; human cell line; liver metastatic model; carbohydrate antigen

The prognosis of patients with extrahepatic bile duct carcinoma is poor despite recent advances in diagnostic imaging and therapeutic techniques (Miyazaki *et al.*, 1987). In many cases, curative surgery is difficult because this carcinoma is usually detected at an advanced stage, spreading invasively along the bile duct and involving the vessels at the time of initial diagnosis. An understanding of the biological nature of this neoplasm is needed to improve the prognosis of these patients. Cultured human cancer cell lines of various organ systems have greatly contributed to the understanding of tumour biology and the definition of tumour-associated antigens. However, extrahepatic bile duct carcinoma cell lines are very rare, and only two such cell lines - SK-ChA-1 (Knuth *et al.*, 1985) and KMBC (Yano *et al.*, 1992) - have been reported. In this paper, we report the establishment of a new human extrahepatic bile duct carcinoma cell line, designated OCUCh-LM1, and an experimental liver metastatic model.

### Materials and methods

### *Origin of cells*

A 61-year-old Japanese man with jaundice was diagnosed as having extrahepatic bile duct carcinoma with multiple liver metastases based on the findings of percutaneous transhepatic cholangiography, computerised tomography and ultrasonography. Serum tumour marker levels on admission were as follows: sialyl Lewis X (SLX), 46.7 U $ml^{-1}$; carbohydrate antigen 19-9 (CA19-9) 225 U $ml^{-1}$; SPan-1 antigen, 140 U $ml^{-1}$; carcinoembryonic antigen (CEA), 1.2 ng $ml^{-1}$; and alpha-fetoprotein (AFP), 7.5 ng $ml^{-1}$. Partial resection of the bile duct, cholecystectomy, choledochojejunostomy and biopsy of a liver metastatic lesion were performed. The cell line described in this paper was derived from this liver biopsy specimen, and the liver metastatic lesion was histologically diagnosed as a well-differentiated adenocarcinoma.

Correspondence: Y-S Chung, First Department of Surgery, Osaka City University Medical School, 1-5-7 Asahimachi, Abenoku, Osaka, 545, Japan
Received 5 July 1994; revised 24 October 1994; accepted 26 October 1994

### *Cell culture*

The specimen was washed in phosphate-buffered saline (PBS) and minced into small pieces with no enzyme present. The cell suspension was filtered through a sterile mesh and centrifuged at 1500 r.p.m. for 5 min, and the cell pellet was then suspended in Dulbecco's modified Eagle medium (DMEM; Bioproducts) supplemented with 10% fetal calf serum (FCS; Commonwealth Serum Laboratories), penicillin (100 IU $ml^{-1}$, Flow Laboratories), streptomycin (100 $\mu g$ $ml^{-1}$, Flow Laboratories), sodium pyruvate (0.5 mM, Bioproducts), and L-glutamine (2 mM, Bioproducts) and seeded into 100 mm dishes. The cells were cultured at 37°C in a humidified atmosphere of 5% carbon dioxide. Cell cultures were subcultured with brief trypsin treatment until detachment of cell islands.

### *Morphological studies*

Cell morphology was observed with a phase-contrast microscope (Nikon TMD). For transmission electron microscopy, a single-cell suspension was centrifuged (1000 r.pm., 5 min) and the pellet was washed three times in PBS. The pellet was fixed for 5 min at 4°C in a 3% glutaralaldehyde solution, and post-fixed for 20 min at 4°C in a 2% osmium tetroxide solution. After embedding of the pellet in Epon, thin sections were cut and stained with uranyl acetate and lead citrate. Electron micrographs were taken with a JEOL 1200X electron microscope (JEOL, Tokyo, Japan).

### *Growth curve*

A suspension of $1.0 \times 10^5$ cells was placed in 35 mm dishes, and the number of cells was counted at intervals of 6-12 h for 5 days with a Coulter counter (Coulter Electronics) (Sanford *et al.*, 1951). The doubling time was determined from the growth curve.

### *DNA analysis*

Flow cytometric analysis of nuclear DNA content of OCUCh-LM1 cells was performed using a FACScan (Becton Dickinson).

*Chromosome study*

Chromosome analysis using G-banding was performed on cells at the 12th passage (Seabright, 1971). OCUCh-LM1 cells were treated with colcemid (Gibco Laboratories) for 2 h at a final concentration of 0.05 $\mu g\ ml^{-1}$. Cells were resuspended in a hypotonic solution (0.075 M potassium chloride). Fixation was performed in Carnoy's fixative. The cells were dropped onto chilled glass slides, stained with Giemsa solution, mounted, and photographed for counting. Twenty cells in metaphase were analysed.

*Tumour-associated antigen secretion*

The secretion of tumour-associated antigens was studied with the supernatants collected from cells cultured for 5 days. The SLX (Fukushi *et al.*, 1985) and CA19-9 (Koprowski *et al.*, 1979) levels of the supernatants were determined by SLX Otsuka kit (Otsuka Assay Laboratories, Tokushima, Japan) and CA19-9 RIA kit (Centocor, Malvern, PA, USA) respectively. The SPan-1 antigen (Chung *et al.*, 1987; Ho *et al.*, 1988) level was determined by sandwich radioimmunoassay (RIA), as described previously (Chung *et al.*, 1987). The CEA (Gold and Freedman, 1965) level was determined by CEA RIABEAD kit (Dainabot, Tokyo, Japan).

*Heterologous transplantation*

Four- to six-week-old female nude mice (Balb/c, *nu/nu*, Clea Japan, Osaka, Japan) were used to examine the tumorigenicity of OCUCh-LM1 cells. A total of $1.0 \times 10^7$ cultured cells suspended in 0.5 ml of PBS were inoculated subcutaneously into the back of each of four nude mice. The mice were sacrificed 8 weeks after inoculation and the reconstituted tumours were used for histological and immunohistochemical studies. All procedures involving animals were conducted in accordance with the UKCCCR guidelines for the welfare of animals in experimental neoplasia.

*Immunohistochemical studies of tumour-associated antigens*

The avidin–biotin–peroxidase complex method (Hsu *et al.*, 1981) was used to identify the expression of SLX, CA19-9, SPan-1 and CEA in the reconstituted tumour in a nude mouse. Specimens fixed in formalin and embedded in paraffin were deparaffinised and treated with 0.03% hydrogen peroxide in methanol for 30 min to block endogenous peroxidase activity. After the sections were rehydrated and washed with PBS, normal bovine serum was applied for 5 min and drained. Primary monoclonal antibodies (FH-6, NS19-9, SPan-1 -antibody and CEA 010) were applied for 30 min. After incubation at room temperature, the sections were washed twice with PBS and incubated for 30 min at room temperature with biotinylated secondary antibody. After two washes, the sections were incubated with avidin–biotin–peroxidase complex for 30 min at room temperature and reacted with diaminobenzidine tetrahydrochloride for 5 min. Finally the sections were counterstained with methyl green and mounted. The anti-sialyl Lewis X antibody (FH6, murine IgM) and anti-SPan-1 antibody (murine 1gM) used were kindly supplied by Otsuka Assay Laboratories and Dainabot respectively. The anti-CA19-9 antibody (NS 19-9, murine IgG) was obtained from Fuji-Rebio and the anti-CEA antibody (CEA 010) was obtained from Mochida.

*Liver metastasis model in nude mice*

Nude mice were anaesthetised with ethyl ether. The abdominal wall was incised, and the spleen was exposed. A total of $1.0 \times 10^6$ OCUCh-LM1 cells suspended in 0.1 ml of PBS were injected into the lower pole of the spleen. Splenectomy was performed after splenic injection, and the abdominal wall and skin were closed with a continuous suture. The mice were sacrificed 8 weeks after the injection. Metastasis to the liver was evaluated as the number of tumour nodules in the liver. Several liver metastases were dissected free of the liver and minced into small pieces with no enzyme present, and the cell suspension was recultured in 10% FCS–DMEM. These cells were used for establishing a highly metastatic cell line. When the cultures became semiconfluent, cells were collected, diluted to $1.0 \times 10^6$ cells 0.1 $ml^{-1}$ and again injected into the spleen of nude mice. This procedure was repeated three times, and the daughter cell lines were designated OCUC-LM1-H1, -H2 and -H3.

### Results

*Establishment of OCUCh-LM1*

A few days after the primary culture, some epithelial cell-like colonies were observed on the bottom of the plastic dishes. When the cultures became confluent, the attached colonies were washed with PBS, trypsinised and replated into plastic dishes. Intermingled fibroblasts gradually decreased in number and finally disappeared after 1 month of primary culture. This cell line grew continuously and has been maintained for over 60 passages over 1 year, and has been designated OCUCh-LM1.

*Morphological studies and growth curve*

Phase-contrast microscopy of OCUCh-LM1 cells disclosed a monolayer cobblestone-like pattern. The OCUCh-LM1 cells had clear cytoplasm and oval nuclei (Figure 1). Ultrastructural analysis of OCUCh-LM1 cells revealed large indented

**Figure 1** Phase-contrast photomicrograph of OCUCh-LM1 cells showing a cobblestone-like pattern.

**Figure 2** Electron micorgraph of OCUCh-LM1 cells, showing large indented nuclei (white arrowhead), junctional complexes (black arrowhead) and numerous microvilli (arrow) on their surfaces.

nuclei. The OCUCh-LM1 cells had junctional complexes between cells and numerous microvilli (Figure 2).

OCUCh-LM1 cells grew rapidly, and the population doubling time for the logarithmic growth phase was 31 h (Figure 3).

*DNA analysis and chromosome study*

DNA analysis of OCUCh-LM1 demonstrated an aneuploidy pattern with a DNA index of 1.76 (Figure 4). Chromsome counts of metaphase cells demonstrated a unimode of 63. The karyotype was human type with abnormal structure (1,2,4,10,11,13,14,15 trisomy, 3p−, 8 monosomy, 12p+, 16q−, 17q+, 17q+, 18, 18, 18, 19p+, 21p+, mar, mar) (Figure 5).

*Tumour-associated antigen secretion*

The levels of tumour-associated antigens secreted into the conditioned medium were as follows: SLX, 32.3 U $ml^{-1}$; CA19-9, 85.0 U $ml^{-1}$; SPan-1, 67.2 U $ml^{-1}$; and CEA <1.0 ng $ml^{-1}$ (Table I). High levels of SLX, CA19-9 and SPan-1 were found in the spent medium.

Figure 3 Growth curve for OCUCh-LM1 cells. The population doubling time for the logarithmic growth phase was 31.0 h.

*Heterologous transplantation*

OCUCh-LM1 cells were tumorigenic in nude mice, and tumours were observed in all four nude mice tested. Inoculation with $1.0 \times 10^7$ OCUCh-LM1 cells resulted in detectable tumours after 7 days. The reconstituted tumours exhibited characteristics similar to the original neoplasm and were classified as well-differentiated adenocarcinomas (Figure 6).

*Immunohistochemical studies of tumour-associated antigens*

Immunohistochemical staining for SLX, CA19-9 and SPan-1 in the reconstituted tumour of a nude mouse was most strongly positive on the cell membrane and not entirely clear in the cytoplasm. However, expression of CEA in this tumour was not seen (Figure 7).

*Liver metastasis model in nude mice*

Eight weeks after splenic injection of OCUCh-LM1 cells, liver metastases were observed in only one of four nude mice. Only one metastatic nodule was present. A daughter line was established from liver metastatic colonies of OCUCh-LM1 and designated OCUCh-LM1-H1. Eight weeks after splenic injection of OCUCh-LM1-H1 cells, liver metastases were

Figure 4 Flow cytometric analysis of nuclear DNA content of OCUCh-LM1 cells: the DNA pattern was aneuploidy and the DNA index was 1.76.

Figure 5 A G-banded karyotype of OCUCh-LM1 cells with a unimode of 63. Arrowheads indicate chromosomes with structural rearrangements.

**Table I** Tumour-associated antigens produced in the spent media of culture of OCUCh-LM1

| *SLX* (*U ml*$^{-1}$) | *CA19-9* (*U ml*$^{-1}$) | *SPan-1* (*U ml*$^{-1}$) | *CEA* (*ng ml*$^{-1}$) |
|---|---|---|---|
| 32.3 | 85 | 67.2 | <1.0 |

**Table II** Production of liver metastasis by OCUCh-LM1 cells injected into the spleen of nude mice

| *Cell lines* | *No. of mice with liver metastasis total* | *No. of liver colonies* |
|---|---|---|
| OCUCh-LM1 | 1 4 | 0.25 ± 0.43 (0-1) |
| OCUCh-ML1-H1 | 4 4 | 13.75 ± 6.61 (3-21)[a] |
| OCUCh-LM1-H2 | 3 3 | 14.33 ± 5.44 (7-20)[a] |
| OCUCh-LM1-H3 | 3 3 | 12.67 ± 4.78 (6-17)[a] |

[a]The increase in incidence of liver metastasis between OCUCh-LM1 and the daughter cell lines (OCUCh-LM1-H1, -H2 and -H3) was highly significant. However, no differences were detected among H1, H2 and H3 in incidence of liver metastasis. The statistical significance of differences in number of liver metastasis was determined using Student's $t$-test.

**Figure 6** Histological findings for the primary lesion (top), the metastatic liver lesion (middle) and the subcutaneous tumour in a nude mouse (bottom). All were well-differentiated adenocarcinomas (original magnification × 200).

observed in all four nude mice tested. The number of metastases of OCUCh-LM1-H1 in the liver averaged 13.75 ± 6.6; for -H2, 14.33 ± 5.44; and for -H3, 12.67 ± 4.78 (Table II, Figure 8). The metastatic nodules were well-differentiated adenocarcinoma, similar to the original neoplasm and the reconstituted tumours in the nude mice (Figure 9).

### Discussion

Advances in cell culture techniques have made it possible to establish a variety of human carcinoma cell lines. However, cell lines originating from biliary carcinomas have rarely been

**Figure 7** Expression of SLX, CA19-9, SPan-1 and CEA in the reconstituted tumour in a nude mouse. SLX, CA19-9 and SPan-1 are expressed on the luminal surface and secretory products, but CEA is negative (original magnification × 200).

**Figure 8** Liver metastasis of OCUCh-LM1-H1 cells at 8 weeks after injection into the spleen of a nude mouse.

**Figure 9** Histological findings for the liver metastatic nodule in a nude mouse demonstrating well-differentiated adenocarcinoma (original magnification × 200).

reported. Only two extrahepatic bile duct carcinoma cell lines (SK-ChA-1 and KMBC) have been reported in the world literature. Since bile duct carcinoma proliferates invasively along the bile duct, it is rich in interstitial stromal cells but has relatively few cancer cells in primary lesions. This may be the reason why the establishment of cell lines from extrahepatic bile duct carcinoma has been very difficult. In fact, SK-ChA-1 was established from a peritoneal effusion resulting from peritoneal dissemination, and KMBC was established from a serially transplanted tumour in nude mice that originated from the tumour at the primary site. In these specimens, there were many cancer cells but few interstitial stroma cells. The OCUCh-LM1 cell line was established from a metastatic liver lesion. Since this metastatic liver lesion had many cancer cells, as did the peritoneal effusion and serially transplanted tumour noted above, it was probably suitable for cell culture.

High levels of SLX, CA19-9 and SPan-1 were found in the conditioned medium of OCUCh-LM1 cells, and positive staining with MAbs against these carbohydrate antigens was observed in all the reconstituted nude mouse tumours. These findings paralleled those for serum tumour marker levels in the original patient. OCUCh-LM1 cells are strongly tumorigenic and induced tumour formation in 100% of nude mice following subcutaneous injection. The reconstituted tumours histologically exhibited characteristics similar to those of both the primary lesion and the metastatic liver lesion, and all tissues were classified as well-differentiated adenocarcinoma. Thus, OCUCh-LM1 cells appear to retain the morphological and functional characteristics of the original human tumour.

The pathogenesis of metastasis begins with the invasion of tissues, blood vessels and or lymphatics by cells originating from a primary tumour. Following their release into the circulation, tumour cells adhere to the capillary bed. After initial adhesion, tumour cells must invade the parenchyma, establish a microenvironment, escape host defence mechanisms and finally grow into secondary tumours. The question of why cancer cells metastasise is one of the most important issues in tumour biology. In spite of the importance of this phenomenon, little is known concerning the pathogenesis of metastatic foci or their relationship to the primary tumour. It is necessary to establish a model of metastasis in order to understand the underlying mechanism. Recently, the nude mouse model of human cancer metastasis has been established (Fidler, 1986; Morikawa *et al.*, 1988). Implantation of human colon and pancreatic carcinoma cells into the spleen of nude mice can result in hepatic metastasis (Giavazzi *et al.*, 1986; Vezeridis *et al.*, 1989, 1990). However, there has been no report of an experimental liver metastatic model with an extrahepatic bile duct carcinoma. We therefore attempted to establish an experimental liver metastatic model with OCUCh-LM1 cells. In this model, the incidence of liver metastasis was only 20%. On the other hand, in the liver metastatic model with daughter cell lines (OCUCh-LM1-H1, -H2 and -H3), the incidence of liver metastasis increased to 100%. These differences in incidence of liver metastasis might be attributable to the clonal selection of tumours during the process of liver metastasis. However, no differences were found in the incidence of liver metastasis and the number of metastatic liver nodules among the OCUCh-LM1-H1, -H2 and -H3 daughter cell lines. These findings suggest that the first procedure was the most important for the acquisition of a high capacity for metastasis. In this established liver metastatic model, we can observe the phenomena after intravasation of tumour cells. Therefore, it can be supposed that the processes of cell adhesion, extravasation and angiogenesis are different in the parental cell line and the daughter cell lines.

OCUCh-LM1 cells exhibit many of the characteristics, particularly the expression of various carbohydrate antigens, of an extrahepatic bile duct carcinoma. Many carbohydrate antigens defined by monoclonal antibodies have been used as cancer-associated antigens and for the preoperative diagnosis of certain cancers. Recently, some carbohydrate antigens have been reported to play a significant role in cancer metastasis. Sialyl Lewis X (Lowe *et al.*, 1990; Phillips *et al.*, 1990, Waiz *et al.*, 1990; Tiemeyer *et al.*, 1991) and sialyl Lewis A (Berg *et al.*, 1991; Takada *et al.*, 1991; Tyrrell *et al.*, 1991) have been shown to serve as ligands for ELAM-1 (endothelial leucocyte adhesion molecule 1), which is expressed on the endothelium, and are probably involved in the process of adhesion between cancer cells and endothelial cells in target organs.

Since OCUCh-LM-1 cells express these carbohydrate antigens and OCUCh-LM1-H1, -H2 and -H3 induced liver metastasis in all of the nude mice tested, OCUCh-LM1 and the experimental liver metastatic model of OCUCh-LM1-H1, -H2 and -H3 appear to be useful for the study of the biological nature of extrahepatic bile duct carcinoma and the relationship between expression of carbohydrate antigens and metastatic potential.

### References

BERG EL, ROBINSON MK, MANSSON O, BUTCHER EC AND MAGNANI JL. (1991). A carbohydrate domain common to both sialyl Lewis[a] and sialyl Lewis[x] is recognized by the endothelial cell leukocyte adhesion molecule ELAM-1. *J. Biol. Chem.*, **266**, 14869-14872.

CHUNG YS, HO JJL, KIM YS, TANAKA H, NAKATA B, HIURA A, MOTOYOSHI H, SATAKE K AND UMEYAMA K. (1987). The detection of human pancreatic cancer-associated antigen in the serum of cancer patients. *Cancer*, **60**, 1636-1643.

FIDLER IJ. (1986). Rationale and methods for the use of nude mice to study the biology and therapy of human cancer metastasis. *Cancer Metastasis Rev.*, **29**, 29-49.

FUKUSHI Y, KANNAGI R, HAKOMORI S, SHEPARD T AND KULANDER BG. (1985). Location and distribution of difucoganglioside in normal and tumor tissues defined by its monoclonal antibody FH6. *Cancer Res.*, **45**, 3711-3717.

GIAVAZZI R, JESSUP JM, CAMPBELL DE, WALKER SM AND FIDLER IJ. (1986). Experimental nude mouse model of human colorectal cancer liver metastases. *J. Natl Cancer Inst.*, **77**, 1303-1308.

GOLD P AND FREEDMAN SO. (1986). Demonstration of tumor-specific antigens in human colon carcinoma by immunological tolerance and absorption techniques. *J. Exp. Med.*, **121**, 439-471.

HO JJL, CHUNG YS, FUJIMOTO Y, BI N, RYAN W, YUAN S, BYRD JC AND KIM YS. (1988). Mucin-like antigen in a human pancreatic cancer cell line identified by murine monoclonal antibodies SPan-1 and YPan-1. *Cancer Res.*, **48**, 3924-3931.

HSU SM, RAINE L AND FANGER H. (1981). Use of avidin-biotin-peroxidase complex (ABC) in immunoperoxidase techniques: a comparison between ABC and unlabeled antibody (PAP) procedures. *J. Histochem.*, **29**, 577-580.

KNUTH A, GABBERT H, DIPPOLD W, KLEIN O, SACHSSE W, BITTER-SUERMANN D, PRELLWITZ W AND MEYER ZUM BUSCHENFELDE KH. (1985). Biliary adenocarcinoma characterization of three new human tumor cell lines. *J. Hepatol.*, **1**, 579-596.

KOPROWSKI H, STEPEWSKI Z AND MITCHELL L. (1979). Colorectal carcinoma antigens detected by hybridoma anti-bodies. *Somatic Cell Genet.*, **5**, 957-972.

LOWE JB, STOOLMAN LM, NAIR RP, LARSEN RD, BERHEND TL AND MARKS RM. (1990). ELAM-1-dependent cell adhesion to vascular endothelium determined by a transfected human fucosyltransferase cDNA. *Cell*, **63**, 475-484.

MIYAZAKI I, KONISHI I AND NAGAKAWA T. (1987). Standard operation for biliary tract cancer and its follow-up results. *Surg. Ther.*, **56**, 443-452.

MORIKAWA K, WALKER SM, JESSUP JM AND FIDLER IJ. (1988). In vivo selection of highly metastatic cells from surgical specimens of different primary human colon carcinoma implanted into nude mice. *Cancer Res.*, **48**, 1943-1948.

PHILLIPS ML, NUDELMAN E, GAETA FCA, PEREZ M, SINGHAL AK, HAKOMORI S AND PAULSON JC. (1990). ELAM-1 mediates cell adhesion by recognition of a carbohydrate ligand, sialyl-LeX. *Science*, **250**, 1130-1132.

SANFORD KK, EARLE WR, EVANS VJ, WALTZ HK AND SHANNON JE. (1951). The measurement of proliferation in tissue cultures by enumeration of cell nuclei. *J. Natl Cancer Inst.*, **II**, 773–775.

SEABRIGHT M. (1971). A rapid banding technique for human chromosomes. *Lancet*, **ii**, 971–972.

TAKADA A, OHMORI K, TAKAHASHI N, TSUYUOKA K, YAGO K, ZENITA K, HASEGAWA A AND KANNAGI R. (1991). Adhesion of human cancer cells to vascular endothelium mediated by a carbohydrate antigen, sialyl Lewis A. *Biochem. Biophys. Res. Commun.*, **179**, 713–719.

TIEMEYER M, SWIEDLER SJ, ISHIHARA M, MORELAND M, SCHWEINRUBER H, HIRTZER P AND BRANDLEY BK. (1991). Carbohydrate ligands for endothelial-leukocyte adhesion molecule 1. *Proc. Natl Acad. Sci. USA*, **88**, 1138–1142.

TYRRELL D, JAMES P, RAO N, FOXALL C, ABBAS S, DAS GUPTA F, NASHED M, HASEGAWA A, KISO M, ASA D, KIDD J AND BRANDLEY BK. (1991). Structural requirement for the carbohydrate ligand of E-selectin. *Proc. Natl Acad. Sci. USA*, **88**, 10372–10376.

VEZERIDIS MP, DOREMUS CM, TIBBETTS LM, TZANAKAKIS G, MEITNER PA. (1989). Experimental metastases from a human pancreatic adenocarcinoma in athymic mice. *Surg. Res. Comm.*, **319**, 313–319.

VEZERIDIS MP, MEITNER PA, TIBBETTS LM, DOREMUS CM, TZANAKAKIS G AND CALABRESI P. (1990). Heterogeneity of potential for hematogenous metastasis in human pancreatic carcinoma. *J. Surg. Res.*, **48**, 51–55.

WAIZ G, ARUFFO A, KOLANUS W, BEVILACQUA M AND SEED B. (1990). Recognition by ELAM-1 of the sialyl-Le X determinant on myeloid and tumor cells. *Science*, **250**, 1132–1135.

YANO H, MARUIWA M, IEMURA A, MIZUGUCHI A AND KOJIRI M. (1992). Establishment and characterization of a new human extrahepatic bile duct carcinoma cell line (KMBC). *Cancer*, **69**, 1664–1673.